# Paper from Wood

**Published:** 1885-02

**Authors:** 


### PAPER FROM WOOD.

The advent of wood pulp wrought a marked change in paper manufacture as soon as this material became known as a substitute, in part, for rags and other stock. It is admitted by nearly all the manufacturers and users of paper that a proper proportion of wood pulp is of great benefit to the sheet, both in giving it "body" and in adapting it for taking ink. A proportion of wood, practically determined, does not impair the strength of the paper, but adds to its value. Chemical wood pulp, while retaining the best fiber, costs double the price of ground pulp, which is now more generally used. Wood is certainly the cheapest of all paper stock, and is the easiest to convert into pulp.

Improvements in the manufacture of pulp, as well as in its application to paper making, are following one another in rapid succession, and at the rate of advancement for the last five years, we may soon expect to see a very respectable sheet of paper made from the natural stick of wood, and by a continuous process, in one establishment. But wood pulp has other uses aside from paper making. Already it has been transformed into barrels, casks, pails and other wooden ware, while boxes, cornices, picture frames and papier-mache articles are successfully made from this material in various parts of the country. In the West it is claimed that the use of weather boarding may be avoided by substituting a covering made of wood pulp, and it is said not only to operate to good advantage for this purpose, but also to work well for doors.

At first it was thought that nothing but poplar, or "popple," as it is sometimes called in New England, would answer for pulp, on account of the peculiar and desirable whiteness which this wood possesses; but to-day we see many other kinds in successful use. Buckeye has been adapted in the West to a considerable extent, and although it is not so strong as poplar, its fiber is very white. In New

England, besides poplar, spruce, pine, chestnut, basswood, fir, hemlock and cedar have been found suitable for various kinds of paper. The difference between the resinous and non-resinous woods is very marked as regards this industry, the latter being adapted for white paper only, while the former serves well for colored.

The Allen machines are arranged to handle all kinds of wood with equal facility, though the different woods grind very differently on the same stones. Willow makes the longest fiber of any wood when thus ground; basswood comes next, and then poplar. Spruce grinds harder and firmer than the former wood. Hemlock, cedar and fir grind about alike, though hemlock is rather the freest in pulping. Maple is quite hard to grind, but its fibre is shorter than either spruce or pine. Poplar and buckeye, when pulped, remain white for a long time, whereas the other woods change their color, birch turning pink, maple purple, and basswood reddish. The aggregate product of ground wood pulp in the United States at the present time is over 200 tons per day.

